# A 58-Year-Old Female with Progressive Cough and Right Shoulder Pain

**DOI:** 10.1155/2016/3049298

**Published:** 2016-10-31

**Authors:** Sanket R. Thakore, Faisal A. Khasawneh

**Affiliations:** ^1^Department of Internal Medicine, Texas Tech University Health Sciences Center, Amarillo, TX, USA; ^2^Section of Infectious Diseases, Department of Internal Medicine, Texas Tech University Health Sciences Center, Amarillo, TX, USA

## Abstract

Cavitary pneumonia in immunocompromised patients is a challenging entity. Establishing accurate diagnosis and starting effective antibiotics are essential steps towards improving outcome. A 58-year-old stem cell transplant patient was admitted to the hospital with necrotizing pneumonia caused by nocardia. The disease progressed despite of aggrieve antimicrobial therapy. Nocardiosis continues to be a difficult disease to diagnose and treat.

## 1. Case Presentation

A 58-year-old female after allogeneic stem cell transplant (SCT) for acute myelogenous leukemia (AML) presented with cough productive of brown sputum and right shoulder pain. Her symptoms progressed over the course of 2 weeks. She did not have fever, chills, or shortness of breath. She denied chest pain, vomiting, or diarrhea and she did not report headache, loss of consciousness, or seizures.

She was diagnosed with AML 2 years ago for which she underwent allogeneic SCT. Thereafter she developed chronic graft versus host disease (cGVHD), which was treated with steroids and mycophenolate mofetil. Her home medications included prednisone, mycophenolate mofetil, and dapsone.

On examination, she was afebrile with a heart rate of 57, blood pressure of 150/74, and oxygen saturation of 93% on room air. Lung examination revealed reduced air entry and rhonchi over the right upper lung zone. Heart exam revealed regular heart sounds without murmurs and her abdomen was soft without tenderness or organomegaly.

**Figure 1:**
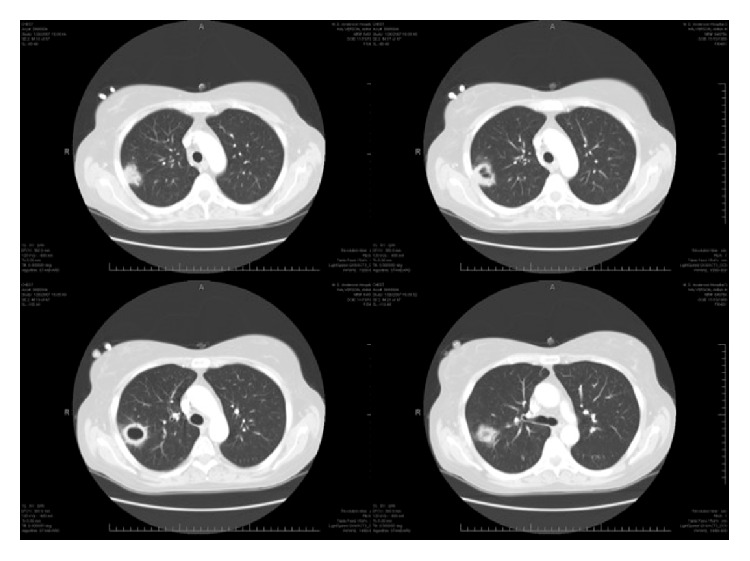
Computed tomography scan sections of the lungs showing right-sided cavitary nodule abutting the chest wall.

Laboratory tests showed white blood cell count of 7.6 × 10^9^ cells/L (88% neutrophils), hemoglobin of 94 g/L, platelets of 34 × 10^9^ cells/L, and creatinine of 70 *μ*mol/L. Chest computed tomography (CT) scan was done and is shown in Figure 1. Bronchoalveolar (BAL) stains and cultures were negative for bacteria, mycobacteria, and fungi. Serum as well as BAL galactomannan and serum cryptococcal antigen were negative. Her prednisone dose was reduced and she was discharged home on ertapenem and voriconazole that were given on empirical basis.

**Figure 2:**
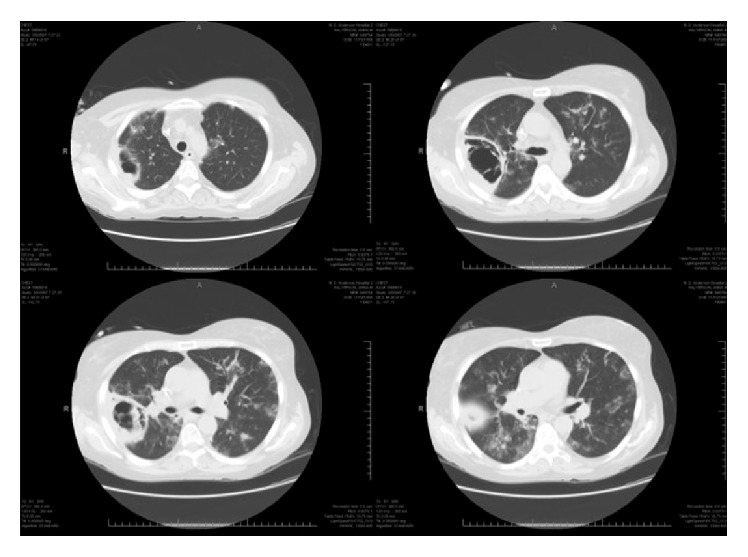
Computed tomography scan sections of the lungs showing progressive enlargement of the right-sided cavitary nodule and the development of new bilateral alveolar opacities.

The patient's symptoms persisted, so a follow-up chest CT scan was done 2 weeks into treatment (Figure 2). She was readmitted to the hospital and started on broad-spectrum antibiotics: linezolid, imipenem, amikacin, and liposomal amphotericin B. Her vital signs on admission were stable with an oxygen saturation of 93% on room air. Labs were within her baseline. A repeated BAL was performed.

What is your diagnosis?

## 2. Diagnosis

BAL stains were negative but bacterial cultures grew few branching gram-positive rods that were identified to be* N. farcinica.* Susceptibility testing was not performed. Liposomal amphotericin B and amikacin were stopped and she continued taking linezolid and imipenem. Trimethoprim/sulfamethoxazole (TMP/SMX) could not be used because she was allergic to sulfa drugs and, unfortunately, sulfa desensitization was not considered. Head CT scan was done and was negative for any brain lesions. Her cough and chest pain worsened and she developed progressive shortness of breath and fevers. Amikacin was added to the regimen without significant improvement in symptoms. She elected to pursue comfort care measures and was discharged to hospice.

## 3. Discussion

Nocardia species are ubiquitous in nature. Nocardia is a gram-positive aerobic filamentous bacterium and, unlike mycobacteria, it has a “beaded” acid-fast appearance. Nocardia can resemble actinomyces species on gram stain; however the latter is anaerobic and does not take acid-fast bacilli stain [1, 2]. Nocardia is known to cause infections in immunocompromised and occasionally immunocompetent patients [3].* N. asteroides complex* is the most commonly encountered and is comprised of* N. abscessus*,* N. cyriacigeorgica*,* N. farcinica*, and* N. nova*. Other nocardia species of medical importance include* N. transvalensis* complex,* N. brasiliensis*, and* N. pseudobrasiliensis* [1].

The numbers of reported* N. farcinica* infections are rising [4]. This is due to technological advancement in diagnostic methods and increasing numbers of immunocompromised hosts. Patients with impaired cell-mediated immunity such as those with lymphoma, leukemia, HIV infection, and organ transplantation and those on long-term steroid or other immunosuppressive therapy are especially at a high risk for this infection [3]. Organ transplantation was cited as one of the major predisposing factors for this infection. The risk of nocardia infection in solid organ transplant recipients is well recognized, but only few cases of nocardia infection after bone marrow transplantation have been reported [5]. Most SCT patients acquire nocardia infection during the first 2 years after transplantation [1].

In nocardiosis the lungs are the most frequently involved organ and the most common portal of entry. Pulmonary nocardiosis can present as an acute, subacute, or chronic infection. The symptoms may include productive or nonproductive cough, shortness of breath, chest pain, hemoptysis, fever, and night sweats. The radiologic manifestations are nonspecific and include nodules with or without cavitation, reticulonodular opacities, and pleural effusion [6]. In light of the above, pulmonary nocardiosis is easily misdiagnosed as bacterial pneumonia, tuberculosis, or other cavitary lung processes [7].

Extrapulmonary nocardiosis is relatively common and occurs through contiguous spread within the thoracic cavity and hematogenous dissemination. The central nervous system (CNS) is the most common extrapulmonary system to be involved, and more than one intracranial abscess can be found [1]. Skin involvement as a primarily or secondarily affected organ has been described. Nocardia bacteremia is uncommon, but cases have been reported. Therefore, all patients with pulmonary nocardiosis should be carefully assessed for disseminated disease by at least a carful skin exam and brain CT scan.

Delay in establishing the correct diagnosis is common due to the nonspecific clinical presentation and the difficulty in cultivating nocardia [1]. Microscopic examination of specimens submitted to the microbiology laboratory is the first step in providing a definitive diagnosis. Gram stain and modified acid-fast stain can give initial clue for the diagnosis meanwhile awaiting culture results. Cultures for nocardia require a minimum of 48–72 h before colonies become evident [8]. Because it requires selective cultures and takes longer than usual, it is important that physicians notify the microbiology laboratory personnel about suspecting this infection [7]. Identification of nocardia isolates at the species level by means of routine phenotypic testing is difficult; however, a simple identification scheme, based on a panel of nine conventional phenotypic and enzymatic tests, has been developed and validated for the rapid identification of the most common nocardia spp. [8]. With modern molecular techniques, such as polymerase chain reaction, restriction enzyme analysis, and 16S ribosomal RNA (rRNA) gene sequencing, faster and easier nocardia identification is possible; however, not all laboratories are equipped with these modern technologies [8]. According to some literature, sequence analysis of the 16S rRNA gene is becoming the gold standard for definitive species identification [9].

The choice of antibiotic therapy depends on the severity and localization of the infection, presence of dissemination, host's immune status, drug interactions and toxicity, and the nocardia species implicated [8]. It is critical to identify nocardia species, as studies have shown significant variability in antibiotic susceptibility among different species [5]. It is also crucial to do antimicrobial susceptibility testing whenever possible. The Clinical and Laboratory Standard Institute has approved standard susceptibility testing and interpretation method by microdilution in cation-supplemented Mueller-Hinton broth [8].

Sulfonamides continue to be the mainstay of therapy for nocardial infections. Sulfamethoxazole, with or without trimethoprim, and sulfisoxazole are commonly used. Ideally, the dosage should be adjusted to achieve peak blood levels of 100–150 mg/dL [5]. Unfortunately, resistance to sulfa drugs, especially among* N. farcinica* and* N. otitidiscaviarum* isolates, has been reported [8].* N farcinica* also has a high degree of resistance to various other antibiotics, especially broad-spectrum cephalosporins [5].

Data from in vitro analyses of antimicrobial susceptibility patterns performed at the Centers for Disease Control and Prevention, Atlanta, GA, revealed that all* N. farcinica* isolates tested were resistant to ampicillin, cefixime, and tobramycin [10]. The majority were also resistant to rifampin (93%), cefamandole (89%), ceftriaxone (86%), erythromycin (86%), and trimethoprim (82%). The isolates were commonly susceptible to imipenem, dapsone, doxycycline, minocycline, and TMP/SMX (<14% resistant). Finally, all isolates were susceptible to amikacin [10]. For patients with life-threatening infection, it seems prudent to initiate therapy with two or three drugs with the aim of switching to oral monotherapy when the patient's condition is stable and the results of in vitro studies are available [11].

Combination therapy with imipenem and cefotaxime, amikacin and TMP/SMX, imipenem and TMP/SMX, amikacin and cefotaxime, or amikacin and imipenem was shown to be superior to single-agent therapy [11]. Some experts are of the opinion that combination of imipenem with amikacin should be started as the first-line therapy for cerebral and pulmonary nocardiosis [1]. An advantage of amikacin and imipenem is their bactericidal activity, compared to the bacteriostatic activity of the sulfonamides [10].

There are no recommendations for prophylaxis against nocardia infection among immunocompromised hosts; however it has been observed that prevalence of nocardia infection is low among advanced HIV patients who are on daily TMP/SMX for* Pneumocystis jiroveci* prophylaxis. Interestingly intermittent TMP/SMX is not as effective in preventing nocardia as daily TMP/SMX [1]. Immunocompetent patients with pulmonary nocardiosis or disseminated nocardiosis outside the CNS should be treated for 6–12 months. In the case of immunocompromised patients, treatment should continue for 1 year and, if possible, the dose of the immune depressant drugs should be reduced [8].

## 4. Conclusion

The diagnosis of pulmonary nocardiosis requires a high index of suspicion, as the presenting symptoms are nonspecific. One should always keep in mind nocardia infection when immunocompromised patients present with pulmonary infection. Physicians should notify the microbiology laboratory when nocardia is suspected.* N. farcinica* infection is potentially lethal because of its tendency to disseminate and its resistance to many antibiotics. Different nocardia species have variable antibiotic susceptibility pattern; this is why identification of nocardia to the species level is crucial. Sequence analysis of the 16S rRNA gene is becoming the gold standard for definitive species identification. It is also important to screen all patients with pulmonary nocardiosis for disseminated infection. It seems prudent to initiate therapy with two or three drugs with the aim of switching to monotherapy when the patient's condition is stable and the results of in vitro studies are available.
